# Development of Autologous Platelet-Rich Plasma Mixed-Microfat as an Advanced Therapy Medicinal Product for Intra-Articular Injection of Radio-Carpal Osteoarthritis: From Validation Data to Preliminary Clinical Results

**DOI:** 10.3390/ijms20051111

**Published:** 2019-03-05

**Authors:** Alice Mayoly, Aurélie Iniesta, Caroline Curvale, Najib Kachouh, Charlotte Jaloux, Julia Eraud, Marie Vogtensperger, Julie Veran, Fanny Grimaud, Elisabeth Jouve, Dominique Casanova, Florence Sabatier, Régis Legré, Jérémy Magalon

**Affiliations:** 1 Department of Hand and Limb Reconstructive Surgery, Assistance Publique–Hôpitaux de Marseille, La Timone University Hospital, 13005 Marseille, France; alice.mayoly@ap-hm.fr (A.M.); aurelie.iniesta@gmail.com (A.I.); caroline.curvale@ap-hm.fr (C.C.); najib.kachouh@ap-hm.fr (N.K.); regis.legre@ap-hm.fr (R.L.); 2 Department of Plastic and Reconstructive Surgery, Assistance Publique–Hôpitaux de Marseille, La Conception University Hospital, 13005 Marseille, France; charlotte.jaloux@ap-hm.fr (C.J.); julia.eraud@ap-hm.fr (J.E.); dominique.casanova@ap-hm.fr (D.C.); 3 Cell therapy department, Assistance Publique–Hôpitaux de Marseille, INSERM, CBT-1409, La Conception University Hospital, Marseille 13005, France; marie.vogtensperger@ap-hm.fr (M.V.); julie.veran@ap-hm.fr (J.V.); fanny.grimaud@ap-hm.fr (F.G.); florence.sabatier@ap-hm.fr (F.S.); 4 Aix-Marseille University, Assistance Publique–Hôpitaux de Marseille, INSERM, Inst Neurosci Syst, Service de Pharmacologie Clinique et Pharmacovigilance, CIC-CPCET, 13005 Marseille, France; elisabeth.jouve@ap-hm.fr; 5 Aix-Marseille University, INSERM, INRA, C2VN, 13005 Marseille, France

**Keywords:** wrist osteoarthritis, microfat, platelet-rich plasma, cell therapy, adipose tissue

## Abstract

Wrist osteoarthritis (OA) is one of the most common conditions encountered by hand surgeons with limited efficacy of non-surgical treatments. The purpose of this study is to describe the Platelet-Rich Plasma (PRP) mixed-microfat biological characteristics of an experimental Advanced Therapy Medicinal Product (ATMP) needed for clinical trial authorization and describe the clinical results obtained from our first three patients 12 months after treatment (NCT03164122). Biological characterization of microfat, PRP and mixture were analysed in vitro according to validated methods. Patients with stage four OA according to the Kellgren Lawrence classification, with failure to conservative treatment and a persistent daily painful condition >40 mm according to the visual analog scale (VAS) were treated. Microfat-PRP ATMP is a product with high platelet purity, conserved viability of stromal vascular fraction cells, chondrogenic differentiation capacity in vitro and high secretion of IL-1Ra anti-inflammatory cytokine. For patients, the only side effect was pain at the adipose tissue harvesting sites. Potential efficacy was observed with a pain decrease of over 50% (per VAS score) and the achievement of minimal clinically important differences for DASH and PRWE functional scores at one year in all three patients. Microfat-PRP ATMP presented a good safety profile after an injection in wrist OA. Efficacy trials are necessary to assess whether this innovative strategy could delay the necessity to perform non-conservative surgery.

## 1. Introduction

Osteoarthritis (OA) of the wrist is one of the most common conditions encountered by hand surgeons. It is a chronic non-inflammatory joint disease characterised by degenerative lesions of the cartilage, which can result in major functional impairment including painful condition, and a loss of strength and motion [1]. Its incidence is constantly increasing due to the ageing population and sport-related traumatic injuries. Most of OA cases are post-traumatic and may be secondary to scapholunate ligament tears leading to carpal disorganization (named scapholunate advanced collapse or SLAC) or bone injury (sequelae of intra-articular distal radius fracture or scaphoid non-union named scaphoid nonunion advanced collapse also known as SNAC). Idiopathic causes such as idiopathic carpal avascular necrosis (Kienböck’s or Preiser’s disease) or congenital wrist abnormalities (Madelung’s deformity) are rare, but can also lead to wrist OA [2,3]. 

The first line therapeutic measures are currently limited to symptomatic treatment: contention by splints, analgesics and anti-inflammatory drugs [3,4] whereas, intra-articular injections of corticosteroids or hyaluronic acid are used in common practice [3] without high levels of evidence. Whereas, their use in other hand joints is still debated [5]. Surgery is indicated after conservative treatment failure. The main objective is to ensure pain relief, while restoring strength and preserving as much wrist motion as possible. Palliative wrist denervation is usually the first step of treatment due to its lesser consequences on wrist mobility. However, it is inefficient in a quarter to more than a third of cases [6,7]. The other surgical alternatives are: first row carpectomy, total or partial wrist arthrodesis and sometimes prosthesis wrist arthroplasty. These palliative procedures require wrist immobilization, intensive post-operative rehabilitation and can only preserve partial function of the wrist [8,9,10,11,12,13]. 

Thus, minimally invasive therapeutic alternative that could delay the use of these heavy and non-conservative surgeries represent a medico-surgical challenge in management of wrist OA after failure of well-conducted medical treatment. Recently, the emergence of biological therapies has led to the evaluation of intra-articular injections of autologous Platelet-Rich Plasma (PRP) in treatment of chondral degenerative diseases with satisfactory results [14,15,16,17,18]. Briefly, activated platelets release growth factors (GF) with beneficial impacts on regenerative and reparative processes. In addition, autologous adipose tissue has gained great interest because it represents a rich and convenient source of cells for regenerative medicine. The stromal vascular fraction (SVF) cells constitute a heterogeneous cell population, including a large proportion of mesenchymal multipotent stem cells (Adipose-derived stem cells, ADSCs), which are located in the adipose tissue vasculature. Thus, the high abundance of adipose tissue within the body, its high surgical accessibility, and the demonstrated multipotency of ADSCs, especially towards the chondrogenic lineage [19,20,21,22,23], highlighted the potential of adipose tissue-derived products for cartilage repair. Among them, microfat is simply obtained through dedicated fat harvesting that uses a multi perforated cannula with holes of 1 mm allowing to harvest smaller lobules of fat (around 600 µm) [24].

We previously described the safety profile of combining autologous microfat and PRP as a mixed regenerative product injected in the carpus or the fetlock joint of sport horses presenting with degenerative joint disease [25]. A significant lameness reduction, together with an early return to compete were also indicative of potential efficacy. However, from a regulatory point of view, the intra-articular use of PRP mixed-microfat was considered by the French National Agency for Medicines and Health Products Safety (Agence Nationale de Sécurité du Médicament (ANSM)) as an Advanced Therapy Medicinal Product (ATMP) according to the directive No. 1394/2007 from the European Parliament and the European Council [26]. This classification is based on the non-homologous use of microfat in the wrist joint. The consequences are that the preparation of the product should comply with Good Manufacturing Practice (GMP), meaning it is relocated in a cell therapy facility, including the achievement of process validation batches with outstanding characterization of the final product, and definition of biological acceptance criteria. For safety concerns, ANSM also imposes a one-month delay between the treatment of each of the three first patients for safety concerns.

Here, we aimed to outline the phases of biological validation of a GMP-compliant PRP mixed-microfat. ATMP performed prior to approval by the French Agency. We also report the preliminary clinical results at 12 months with the follow-up obtained from the first three patients enrolled in a clinical trial evaluating PRP mixed-microfat in wrist OA.

## 2. Results

### 2.1. Process Validation Batches

In the biological data characterising the experimental PRP, microfat and the mixed ATMP are obtained from the process validation batches. As summarised in Table 1A–C, the PRP obtained was a very pure PRP according to the DEPA (Dose Efficiency Purity Activation) classification [27] with a mean ± SD of 97.4 ± 0.2 % platelets purity at the end of the preparation. Increase factor in platelet concentration compared to whole blood was 2.0 ± 0.4. Platelet aggregation in response to different inductors was considered effective for all conditions tested. All PRP preparations were free of microorganisms, and presented stable biological characteristics at 3 h after the end of the preparation. Production of microfat resulted in a macroscopically pure product with less than 10% of oily or bloody phase with stable results at 3 h after the end of the production. 

**Figure 1:**
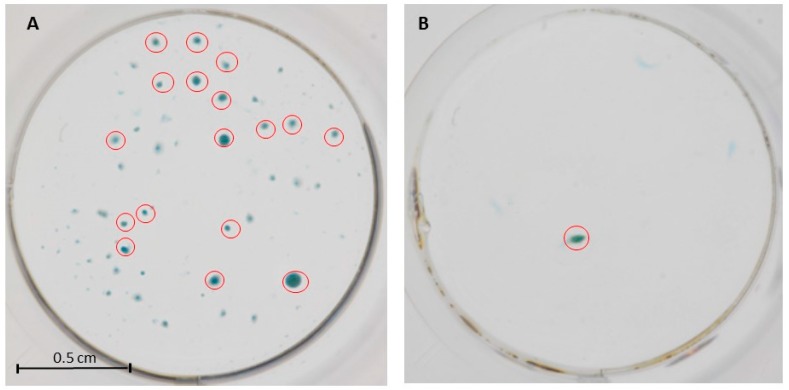
Macroscopic aspect of chondrocytes micromass obtained from adipose-derived stem cells (ADSCs) derived microfat-PRP in the presence of chondrogenic differentiation media (**A**) or control media (**B**). Red circles represent micromass with area > 0.05 mm^2^.

PRP mixed-microfat manufacturing lead to a product containing 1.3 ± 0.6 million viable nucleated Stromal Vascular Fraction (SVF) cells with a mean ± SD viability of 75.7 ± 1.3 %. ADSCs obtained from the mixture were able to differentiate in chondrocytes under specific culture conditions compared to control (Figure 1). Results of stability assays conducted at 3 h on PRP and microfat were similar to t0 data. Finally, PRP mixed-microfat release detectable quantity of regenerative growth factors (VEGF, PDGF, FGF2) whereas, only TGF-β1 was not. Interestingly, IL-1Ra/IL1-β ratio presented a mean ± SD of 443.3 ± 348.0 suggesting a potential anti-inflammatory effect.

**Figure 2:**
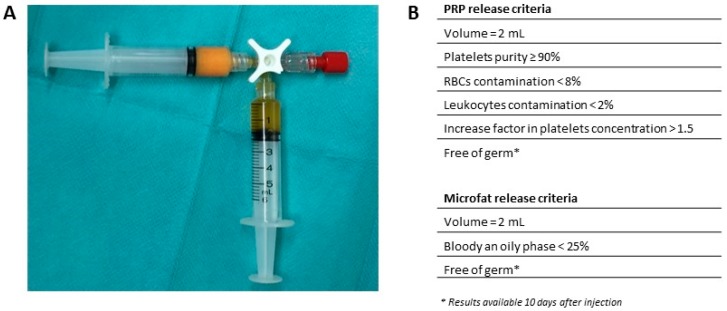
(**A**) Microfat-PRP investigational Advanced Therapy Medicinal Product (ATMP) delivered to the operating room for intra articular injection in radio-carpal joint (without labelling). (**B**) Release criteria from PRP and microfat checked by the responsible pharmacist from the ATMP manufacturing Department before delivery to the operating room.

The final investigational product used for the clinical trial was then defined as two interconnected 5 mL syringes containing 2 mL of microfat and 2 mL of PRP that had to be injected within 3 h after the end of manufacturing. Aspect of the final product delivered to the operating room and release criteria from microfat and PRP were defined and validated by ANSM according to the results of process validation batches and are listed in Figure 2.

### 2.2. Preliminary Data from Clinical Trials

The first three patients were treated between June and August 2017 respecting a one-month delay between each patient, as required by ANSM. Characteristics of patients and injected products are described in Table 2. 

**Figure 3:**
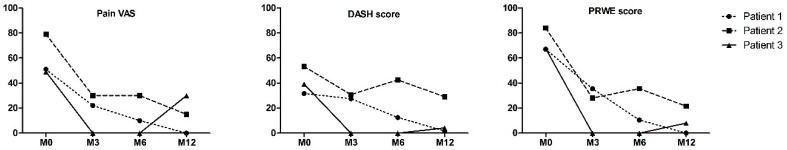
Evolution of pain according to Visual Analog Scale (VAS), Disabilities of the Arm and Shoulder (DASH) and Patient-Rated Wrist Evaluation (PRWE) scores at baseline, and months 3, 6 and 12 for patients 1, 2 and 3.

There was 2.7 to 4 mL of mixture injected with a platelet dose around 700 million and a platelet purity over 95%. Absence of pain was observed during injection (Visual Analog Scale: 0). All patients were able to get back to their regular activities one week after injection. No serious adverse event was recorded in these three patients. In addition, there was no infectious complication observed. The only reported side effect was pain at the sites of the adipose tissue harvesting, which was systematically reported at day seven in a postoperative visit and was relieved by grade 1 analgesics and completely disappeared after one month. All three patients presented with at least a 50% decrease in pain compared to baseline at three months. This effect was maintained until one year after the injection, except for patient three. Disabilities of the Arm and Shoulder (DASH) improvement reached 11 points, corresponding to the minimal clinically important difference (MCID) [28] for patient two and patient three at three months and for all patients at six months. Patient-Rated Wrist Evaluation (PRWE) improvement reached 14 points corresponding to MCID [29] since the third month after treatment for all patients. These observations were stable until the last visit at one year. No significant improvement was observed in grip strength and wrist range of motion during the follow-up. All three patients were either satisfied or very satisfied after the procedure at all follow-up. Figure 3 summarises the results of the pain Visual Analog Scale (VAS), DASH and PRWE scores up to one year.

## 3. Discussion

To our knowledge, this study is the first to report the use of a combined microfat-PRP product as ATMP for joint injection in humans. Indeed, an introduction of adipose tissue in the joint was considered as an ATMP, according to European regulation No. 1394/2007 due to the non-homologous use of adipose tissue in the wrist joint. Thus, a set of accurate biological and pharmaceutical characterisations of the final injected product was conducted, as requested by the regulatory French Agency ANSM prior to any clinical trial authorization. Testing of the process validation batches attested that the PRP mixed-microfat is a sterile product with limited contamination in Red Blood Cells (RBCs) and leukocytes, preserved viability of SVF cells, chondrogenic differentiation ability in vitro and high secretion of IL1Ra anti-inflammatory cytokine. Characteristics of the product were stable upon 3 h after the end of the manufacturing. 

These data allowed to obtain a clinical trial authorization for a phase I study with the constraint to wait one month during the treatments of each of the three first patients for safety reason. The only reported side effect was pain at the site of adipose tissue harvesting. Clinical results achieved MCID for DASH and PRWE scores in all patients one year after injection. MCID is not clearly defined in the literature for VAS scale, which measures pain for wrist OA. That said, VAS scale MCID for surgical treatment of shoulder diseases is between 14 and 30 mm [30,31]. Interestingly, the three patients reached a final improvement at one year of 51, 64 and 19 mm, respectively. The profile of patient three is a matter of discussion, as he presented with an impressive improvement in pain and function up until six months with a slight impairment in pain at one year. In contrast, patient 1 and 2 showed progressive improvement up until one year after treatment. This was explained by a pain wrist crisis in the weeks before the one-year evaluation. However, these subjective assessments were not supported by the objective improvement in measures of strength or range of motion, and should therefore be interpreted carefully.

Absence of adverse event is consistent with other published studies in the literature. Indeed, no infectious or neoplasic complications related to the intra-articular injection of cell-based products (including PRP, cultured ADSCs and/or minimally processed adipose tissue), has been reported to date [15,32,33]. However, very few studies have been designed to address the upper limb, and mainly concern the thumb carpometacarpal joint. Autologous intra-articular fat injection has been studied for the treatment of the thumb carpometacarpal joint OA in two studies. Herold et al. assessed the efficacy of the intra-articular injection of centrifuged adipose tissue in the treatment of rhizarthrosis. They had significantly less pain and a better DASH score at all postoperative time points up until the 12-month follow-up, when they analysed all of the patients together. However, severe patients (grade 4) only presented a slight improvement in DASH score from 57 ± 23 to 51 ± 31 at 12 months with a near return to preoperative value for VAS pain [34]. Kemper et al. [35] assessed the efficacy of surgical arthroscopic debridement combined with autologous fat injections in the treatment of early stages of thumb carpometacarpal osteoarthritis. They showed pain relief at rest, and under stress and an improvement of the QuickDASH score after three to six months, which kept improving over two years. These approaches differ from our procedure, as we used a dedicated harvesting device to obtain small lobules of adipose tissue, also called “microfat” and presenting greater trophic and regenerative qualities than adipose tissue harvested according to “standard” technique [36]. Furthermore, none of these studies have associated PRP to improve the outcomes.

From a regulatory point of view, the preparation and injection of adipose tissue within these two studies were performed in the same surgical procedure, which is simpler and faster than our ATMP procedure. However, the strict application of European Regulation No. 1394/2007 [26] or from the corresponding Food and Drug Administration guidance [37] considered that “regenerating or promoting the regeneration of cartilage or tendon is not a basic function of adipose tissue which is generally not considered a homologous use”. This question is still under debate, as some countries believe the entire procedure can be performed routinely in the operating room and more precision and harmonisation from national and international governing bodies are urgently needed. Nevertheless, the manufacturing of our innovative therapy remains simple, conservative, and minimally invasive and can be performed in a one-day surgery including two consecutive surgeries under local anesthesia. As ATMP production must occur in accordance with the pharmaceutical industry GMP, production costs have increased. However, we were aware that the microfat-PRP ATMP had to be manufactured within a few hours, to anticipate potential sustainability for public institutions in the future. This point should be considered from a cost-effectiveness perspective.

Regarding the mechanism of action, the association of microfat and PRP could be interesting in order to potentiate trophic and regenerative effects on damaged cartilage sites. From a theoretical point of view, the combination of these two products, which are respectively rich in autologous multipotent stem cells and growth factors, aims to create an optimal environment for cartilage cells regeneration. Furthermore, microfat could play the role of scaffold limiting PRP resorption [38,39]. However, this regenerative potential could only be confirmed by the use of appropriate magnetic resonance imaging of the wrist.

The major limitation of this study is the absence of a control group, which is an important weakness knowing the substantial placebo effect in arthrosis care [40]. Furthermore, due to the low number of patients assessed (only three patients), no statistical analysis was conducted.

## 4. Material and Methods

### 4.1. Obtention of Human Tissues and Regulatory Approval

For the process of validation batches, human peripheral blood and abdominal adipose tissue were harvested from three healthy volunteers (42, 33 and 31-year-old females who were not taking any drugs) during esthetic liposuction and after the collection of written informed consent collection. 

The clinical trial was approved by the ethics committee (authorization for AMIPREP trial #16-65 from Comité de Protection des Personnes Sud Méditerranée #1 received 22nd of August 2016) and the ANSM (authorization for AMIPREP trial #160879A-12 received 28^th^ of April 2017) and registered in Clinicaltrials.gov website (NCT03164122 and EudraCT #2016-002648-18). All experiments were conducted in the Cell Therapy facility (authorization #ETI/14/O/005) of our university hospital in compliance with Good Manufacturing Practice.

### 4.2. PRP Preparation

After seven steps of skin decontamination (antiseptic foaming solution, rinsing with sterile water, drying, antiseptic foaming solution, rinsing with sterile water, drying and alcoholic dermal antiseptic), 18 mL of blood was collected by venipuncture using a 21-gauge needle filling one 20 mL syringe containing 2 mL of ACD-A (Proteal, Barcelona, Spain). The blood was transferred into the Orthopras 20+ ^®^ device (Proteal, Barcelona, Spain) before centrifugation using the Omnigrafter III ^®^ (Proteal, Barcelona, Spain) at 3200 rpm over 10 min. The PRP was recovered using the Push Out system and 2 mL of PRP was sampled in a 5 mL syringe. 

For process validation batches, the following analyses were performed on the PRP: cell counting, platelet aggregation test and microbiological assay at t = 0 (immediately after manufacturing) and t = 3 h (claimed shelf life with storage at 18–24°C). 

Cell counting and microbiological assay were performed on clinical trial PRP batches.

#### 4.2.1. Blood and PRP Cell Counting

300 µL from whole blood and each PRP preparation were sampled to determine platelets, leukocytes and red blood cells counts using automated haematology blood cell analyzers Sysmex XN-10 (Sysmex, Kobe, Japan) in accordance with recently published guidelines [41].

#### 4.2.2. Platelets Aggregation Test

Platelets aggregation was assessed by light transmittance aggregometry (LTA) on a four-channel APACT 4004 aggregometer (Elitech, France). To obtain homologous Platelet-Poor Plasma (PPP) as negative control, 1.8 mL of PRP was centrifuged at 2500× *g* during 10 min. Maximal (peak) platelet aggregation (%) induced by ADP 5 µM, ADP 2.5 µM, collagen 3.3 µg/mL, ristocetin 1.25 mg/mL, arachidonic acid 0.5 mg/mL was measured in PRP. 

### 4.3. Microfat Preparation

After seven steps of skin decontamination, harvesting the adipose tissue was performed either in the abdominal area (process validation batches) or in the subcutaneous adipose area of the inner side of the knees (clinical trial batches) after local anesthesia. The microfat harvesting technique was performed using a Hapifat^®^ kit (Benew Medical, Melesse, France). Briefly, a St’rim cannula was both connected to a 10 mL syringe and a purification Puregraft 50^®^ bag through a Fat Lock System^®^. Adipose tissue was then purified two times using 1:1 rinsing with saline solution [42] allowing for the elimination of fluid excess, lipid phase, blood cells, and fragments through filtration by the Puregraft bag membrane. Finally, 2 mL of microfat was sampled in a 5 mL syringe. 

For process validation batches, following analyses were performed on microfat: assessment of macroscopic aspect and microbiological assay at t = 0 and t = 3 h. 

For clinical trial batches, following analyses were performed: assessment of macroscopic aspect and microbiological assay.

#### 4.3.1. Macroscopic Assessment of Microfat

The 5 mL syringe containing 2 mL of microfat was placed vertically allowing sedimentation of oily and bloody phases, considered as contaminants. Quantification was performed visually using syringe graduations by an experimented technician. Contamination lower than 25% of the final volume (0.5 mL) in oil and/or blood was expected.

#### 4.3.2. Microbiological Assay

250 µL of PRP or microfat were sampled in Bactec culture bottles (Peds Plus Aerobic/F and Plus Anaerobic/F culture vials, containing each 40 mL of medium). The Bactec method (Becton Dickinson, Sparks, MD, USA) uses a computer-controlled incubation/detection system. The media used contained proprietary factors designed to inactivate a wide variety of antibacterial and antifungal agents [43]. Bactec culture bottles were incubated at 37 °C for a total of 10 days, and automated readings were taken every 10 min. Detection of organisms resulted in an audible alarm and automatic recording of time to detection. 

### 4.4. Microfat-PRP Mixture

The two 5 mL syringes were connected and mixed by gently shaking the two syringes back and forth ten times to obtain a final product of 4 mL of a homogeneous mixed product.

For process validation batches, following the analyses performed on Microfat-PRP: SVF viability and cell content, ability to differentiate in chondrocytes, GF release. 

For clinical trial batches, following analyses were performed: GF release.

#### 4.4.1. Stromal Vascular Fraction (SVF) Extraction

From 2 to 4 mL of mixture were sampled for SVF extraction. SVF was purified from the Microfat-PRP mixture by collagenase digestion (0.25 U/mL, NB5, Heideberg, Germany) at 37 °C with 5% CO_2_ for 45 min. Total viable nucleated cell recovery and cell viability were determined using the Nucleocounter NC100 instrument (ChemoMetec, Denmark). 

#### 4.4.2. Chondrocytes Differentiation

ADSCs were isolated following SVF cells plating in a 25 cm^2^ flask in the following proliferation media (45% DMEM / 45% HAMS-F12/ 10% fetal bovine serum (Gibco, Thermo Fisher Scientific, Waltham, MA, USA)), GlutaMAX^®^ (100×, Gibco) gentamicin (Panpharma Luitré, France) penicillin G (Panpharma Luitré, France), fungizone (Bristol-Myers Squibb, New York, NY, USA) for ADSCs proliferation. At 80% confluence, ADSCs were trypsined and plated in 24 well-plate in either a chondrocyte differentiation media (StemPro^®^, Thermo Fisher Scientific, Waltham, MA, USA) or proliferation media (considered as control media) at the concentration of 80,000 cells/5 µL. At 10 days, cells were washed using DPBS Ca++/Mg++-free medium (Life Technologies, Carlsbad, California, USA), coloured with 0.015% Alcian Blue 8GX (Sigma-Aldrich, Saint-Quentin-Fallavier, France) to stain glycosaminoglycan cartilage and fixed in 60% ethanol/40% acetic acid to observe chondrocytes micromass. Standardized photographs were taken using a Canon Eos 50D (Canon, Tokyo, Japan) with a 100 mm F2.8 Macro lens. All micromass area ≥0.05 mm^2^ were quantified using NIH ImageJ software and compared with the control group.

#### 4.4.3. Microfat-PRP Growth Factors Release Measurement

The microfat-PRP mixture (500 μL) was placed in a 48-well collagen coated plate (CellAffix, APSciences, Columbia, MD, USA) and incubated for 30 min at 37°C with 5% CO_2_ to allow contact with collagen. After this incubation, 500 μL of non supplemented DMEM (Gibco, Thermo Fisher Scientific, Waltham, MA, USA) was added to the mixture and incubated for 24 h at 37 °C with 5% CO_2_. After 24 h the samples were centrifuged (Multifuge Heraus 3 S-R centrifuge, Thermo Scientific, Indianapolis, IN) at 1500 rpm for 5 min to remove microfat, and samples were stored at −80 °C until used for analysis. A combination of 6 cytokines and growth factors (Vascular Endothelial Growth Factor, Interleukin-1 Receptor antagonist, Fibroblast Growth Factor 2, Interleukin-1β, Transforming Growth Factor β1, Platelet Derived Growth Factor AA-BB or AB-BB) were measured using a Magpix instrument (Luminex xMAP Technology, Luminex Inc., Austin, TX, USA) allowing simultaneous measurement of the different analytes in small sample volume.

### 4.5. Patients

According to the Kellgren and Lawrence classification [44] and with the informed written patient consent, males and females between 18 to 75 years of age who suffered from radio-carpal OA stage four were enrolled. OA resulted from post-traumatic malunion of an articular distal radius fracture or SLAC or SNAC. Only patients that failed conservative treatments (analgesics, anti-inflammatory drugs, splinting, and physiotherapy), defined as having a persistent daily painful condition > 40 mm according to VAS, and that would have otherwise been a candidate to surgery were included. 

#### 4.5.1. Surgical Procedure and Injection

Operative procedure included two consecutive surgical steps performed under local anesthesia in the same day. The first step consisted of lipoaspiration for microfat and harvesting the blood sample. Lipoaspirate and the blood sample were transported in sterile bags to the authorized ATMP manufacturing Unit. Microfat and PRP were prepared as described above and two interconnected syringes were sent back to the operating room within 2 h after harvesting. The second step consisted of the injection of PRP mixed-microfat. Before the intra-articular injection, microfat and PRP were pooled together by making ten transfers between the two connected syringes. After local anesthesia subcutaneously on the dorsal side of the wrist with xylocaine (10 mg/mL) without adrenaline, 4 mL of microfat and PRP was injected using a 16 gauge needle into the radio-carpal joint under X-ray guidance (Ziehm Solo FD, Ziehm Imaging GmbH, Nürnberg, Deutschland). An analgesic immobilization by a specific cast involving compression, contention and cryotherapy (IGLOO^®^) was immediately put in place for seven days. 

#### 4.5.2. Clinical Assessment

The primary endpoint was the safety of the treatment evaluated by the occurrence of adverse events up to one month after the injection (visits at Day 7 and Month 1). Secondary endpoints were the following criteria at 3, 6 and 12 months: subjective pain rating according to VAS (0–100 mm), functional evaluation according to the DASH and the PRWE scores, objective wrist strength measured by dynamometry, and the objective wrist range of motion measured using a goniometer. Patient’s satisfaction was rated on a 5-level scale: Not satisfied, Few Satisfied, Moderately Satisfied, Satisfied, and Very satisfied.

### 4.6. Statistical Analysis

Data are presented as mean ± standard deviation except for microbiological assay (presence/free of germ), chondrocytes differentiation (number of micromass formed compared to control), macroscopic aspect of the microfat (presence/absence of contaminants > 25%). The increase factor in platelets was obtained by dividing the concentration of platelets in PRP by the concentration in whole blood. Doses of platelets in PRP were obtained by multiplying the volume of PRP injected by the corresponding concentration. Platelets purity was obtained by dividing the quantity of platelets in the PRP by the sum of platelets, red blood cells and leukocytes. No statistical analysis was performed due to the low number of samples or patients described in the study.

## 5. Conclusion

The present report demonstrates the feasibility to manufacture, control, and inject a PRP mixed-microfat investigational ATMP and its short-term good tolerance for wrist OA. However, only a greater number of patients documented will improve the safety of the procedure. If this endpoint is met, efficacy trials could then be implemented to define whether this non-invasive strategy can be proposed in selected patients with a history of wrist OA with drug-refractory failure, and be advantageous compared to invasive and non-conservative surgical wrist procedures.

## Figures and Tables

**Table 1:** Biological characteristics of PRP (A), microfat (B), and PRP mixed-microfat (C) obtained from the analysis of GMP compliant process validation batches.

**(A)**	**T0**	**T + 3 h**
**Platelets Purity (%)**	97.4 ± 0.2	97.4 ± 0.1
**RBCs Contamination (%)**	2.5 ± 0.1	2.5 ± 0.1
**Leukocytes Contamination (%)**	0.04 ± 0.04	0.03 ± 0.03
**Potential Dose of Injected Platelets (10^6^/ 2 mL)**	775.3 ± 35.8	766.7 ± 25.8
**Increase Factor in Platelets**	2.0 ± 0.4	2.0 ± 0.4
**Platelets Aggregation Response (%)**		
ADP 5 µM	74.2 ± 9.8	69.0 ± 2.6
ADP 2.5 µM	55.5 ± 7.5	47.8 ± 1.9
Arachidonic Acid 0.5 mg/ mL	91.6 ± 3.5	87.5 ± 1.6
Collagen 3.3 µg/mL	90.1 ± 2.9	91.1 ± 2.4
Ristocetin 1.25 mg/mL	97.7 ± 2.5	96.9 ± 4.0
**Microbiological Assay**	Free of germ (3/3)	Free of germ (3/3)
**(B)**	**T0**	**T + 3 h**
**Macroscopical Aspect**	Absence of blood and oily phase (3/3)	Absence of blood and oily phase (3/3)
**Microbiological Assay**	Free of germ (3/3)	Free of germ (3/3)
**(C)**	**T0**	-
**SVF Viability (%)**	75.7 ± 1.3	-
**SVF Viable Nucleated Cells (x10^6^ / 4 mL)**	1.3 ± 0.6	-
Chondrocytes Differentiation * (Micromass Formed/Control Group)	17/1 10/0	-
**GF Release (pg/mL)**		
VEGF	22.0 ± 30.1	-
PDGF	394.0 ± 128.1	-
FGF2	171.8 ± 83.1	-
TGF-β1	ND	-
IL1-β	0.2 ± 0.1	-
IL-1RA	82.0 ± 73.3	-
IL-1RA/IL1-β ratio	443.3 ± 348.0	-

Results are expressed in mean ± SD except for microbiological assay, macroscopical aspect, and chondrocytes differentiation. Red Blood Cells, (RBCs); Growth Factors, (GF); Stromal Vascular Fraction (SVF) *performed only on two samples.

**Table 2:** Patients and injected products characteristics obtained from the first three patients treated with microfat-PRP ATMP.

	Patient 1	Patient 2	Patient 3
Gender	Female	Female	Male
Age, years	65	62	59
Dominant hand	Right	Right	Right
Injured wrist	Left	Left	Left
Etiology	Radius fracture	SLAC	SLAC
Kellgren Lawrence grade	4	4	4
Seat(s) of osteoarthritis	RC	RC and IC	RC
Volume of microfat PRP injected (mL)	2.7	4.0	3.8
Dose of injected platelets (10^6^)	720.9	674.0	708.7
Platelets purity (%)	96.0	94.2	97.4
PRP microbiological assay	Free of germ	Free of germ	Free of germ
Microfat microbiological assay	Free of germ	Free of germ	Free of germ

R, Radiocarpal; IC, Intracarpal; SLAC, scapholunate advanced collapse.
